# In-theatre demonstration of laparoscopic retroperitoneal anatomy as an educational tool for final-year MBBS students: a novel quasi randomized experiment

**DOI:** 10.1016/j.xagr.2025.100572

**Published:** 2025-10-09

**Authors:** Rumi Bhattacharjee, Sangita Pandey, Manisha Jana, Jaishree Ganjiwale, Nitin Raithatha, Somashekhar Nimbalkar

**Affiliations:** 1 Obstetrics and Gynaecology, Pramukhswami medical college (Bhattacharjee, Pandey, Jana, and Raithatha), Bhaikaka university, Anand, Gujarat, India; 2 Central Research Services, Pramukhswami medical college (Ganjiwale), Bhaikaka university, Anand, Gujarat, India; 3 Neonatology, Pramukhswami medical college (Nimbalkar), Bhaikaka university, Anand, Gujarat, India

**Keywords:** educational tool, multimedia technologies, retroperitoneal anatomy, student-oriented, traditional classroom teaching, uterine artery occlusion

## Abstract

**Introduction:**

Traditional methods of teaching anatomical landmarks and their clinical and surgical significance often exhibit low retention rates. Medical educators have consistently sought to integrate innovative pedagogical approaches in response to evolving educational needs. The advent of multimedia technologies has significantly enhanced engagement and retention in learning, thus promoting a deeper contextual understanding in various clinical settings.

**Aims:**

To compare the effectiveness of laparoscopic interactive demonstration of retroperitoneal vascular anatomy against traditional classroom teaching among medical students concerning knowledge acquisition, memory consolidation, and their perception of the teaching method.

**Design:**

A prospective single-center, two-arm, Quasi-randomized Educational Intervention Trial (allocation ratio 1:1) assigned 152 final-year undergraduate medical students to either the intervention (live interactive schooling) or the control group (conventional classroom teaching). The primary outcome measured was an enhancement in fundamental pelvic retroperitoneal anatomy, which was assessed by administering pre- and post-test questionnaires of equal difficulty. Feedback forms were used to compare the acceptability and perception of the novel and traditional methods. Statistical analysis was performed using STRATA 14.4, paired t-tests for pre- and post-test comparisons, and Likert scale responses for feedback analysis.

**Results:**

Both groups had comparable mean pretest scores. Post-test, the novel-method group demonstrated a significantly greater improvement (8.67±2.81) than the conventional group (5.74±3.18), with a mean difference of 2.93 (95% CI: 1.96–3.90; *P*<.001) and a large effect size (Cohen’s d=0.98), which reflected the principal outcome measure. At six-month follow-up, performance declined in both groups (*P*=.064). Student feedback indicated greater enthusiasm and acceptability for the novel approach.

**Conclusion:**

Live interactive educational techniques were found to be effective, acceptable, and more student-oriented than traditional teaching-learning methods.

**Trial Registration:**

Clinical Trials Registry-India, CTRI/2024/02/062476

URL: https://ctri.nic.in/.

**AJOG Global Reports at a Glance**

**Why was the study conducted?**

Advances in technology have revolutionized surgical education, underscoring the need for innovative platforms that foster the mastery of complex skills. This study investigates the impact of real-time, in-theatre interactive demonstration of laparoscopic retroperitoneal anatomy on the learning outcomes of final-year MBBS students.

**Key findings?**

The results suggest that engaging students in a dynamic surgical setting significantly enhances their anatomical understanding and clinical relevance. The intervention group showed a greater mean improvement in post-test compared to the conventional group (*P*<.001). Feedback showed greater student acceptability and enthusiasm for the novel method over the traditional approach.

**What does this add to what is known?**

While several studies highlight the value of live-streamed surgical procedures, to our knowledge, none have evaluated the effectiveness of live retroperitoneal anatomy demonstrations for undergraduates.

## Introduction

The application of different tools to deliver information to learners to enhance their understanding of concepts can be attributed to the progress of technology over the years.

Hysterectomy is one of the most frequently performed gynecological procedures. Ever since the first minimally invasive procedures were heralded in the late 20^th^ century, the art of endoscopy has been redefined and remodeled.

Surgical knowledge and skills, including hysterectomy, are integral to medical teaching for undergraduates and postgraduates. In addition to the knowledge gained from the text, the conventional teaching method has been to observe /assist /perform the procedure under supervision. Technological application in surgical education has the potential to develop individualized schooling. Many multimedia tools, such as prerecorded videos, photographs, online live surgeries, and live surgical telecasts during workshops and conferences, have been deployed for ongoing learning for health professionals. However, minimal efforts have been directed towards the enhancement of knowledge of undergraduate and postgraduate students in this regard.

**Figure 1:**
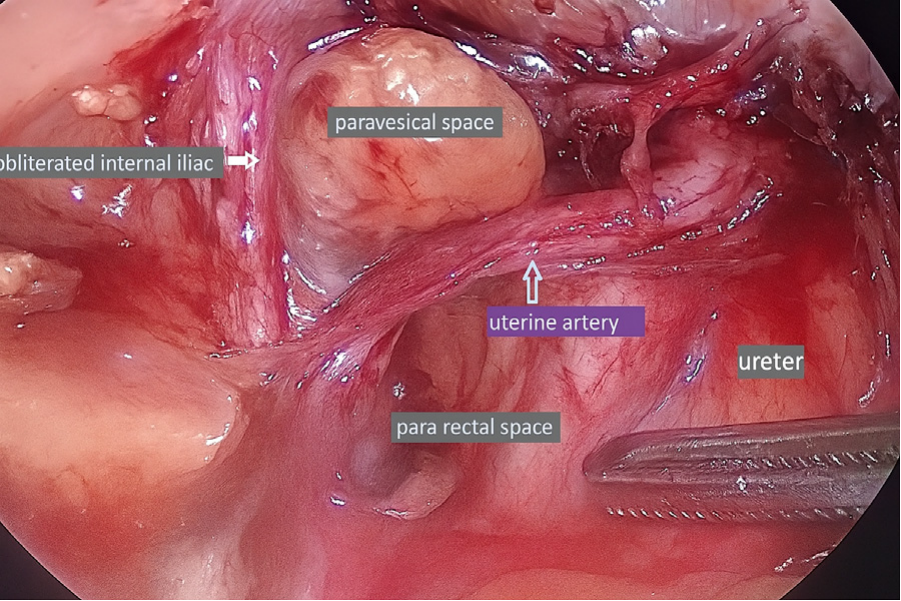
Retroperitoneal spaces with the ureter.

The dissection of the retroperitoneal space and subsequent ligation of the internal iliac or uterine artery necessitates considerable expertise and is best acquired through hands-on experience. This skill is considered essential for postgraduates, as the ligation of the internal iliac arteries can be a lifesaving intervention in cases of intractable postpartum hemorrhage (PPH), a major contributor to maternal mortality and morbidity worldwide. Students cannot easily assimilate the intricate process of ligating the internal iliac artery and /or the uterine artery at its origin, especially in the high-pressure environment of an emergency surgical management of PPH. The pelvic retroperitoneum contains key vascular, urological, lymphatic, and neural structures essential for safe dissection. At the pelvic brim, the common iliac arteries divide into external and internal branches; the external iliac vessels continue as femoral, while the internal iliac supply pelvic organs. The uterine artery, arising from the anterior division, crosses above the ureter (“water under the bridge”), with venous drainage forming extensive pelvic plexuses. The ureter enters the pelvis at the iliac bifurcation, coursing within the broad ligament. Lymphatic drainage parallels the iliac vessels. Surgical access is facilitated by avascular planes—the paravesical space (between bladder and pelvic sidewall) and the pararectal space (between ureter/uterine artery and internal iliac vessels). (Figure 1) Hypogastric nerves, running parallel to the ureter and forming the inferior hypogastric plexus, must be preserved during nerve-sparing procedures.^1^ Fedortsov et al argue that live surgery broadcasts offer greater educational value than recorded videos, as they enhance engagement and understanding through real-time interactivity and spontaneity.^2^

Bhattacharjee et al. In-theatre demonstration of laparoscopic retroperitoneal anatomy as an educational tool for final-year MBBS students. Am J Obstet Gynecol 2025.

Multimedia technology combines verbal instructions with visual imagery to enhance expression and comprehension while providing educators with valuable insights into students' learning progress.^3^ In surgical teaching, this approach facilitates direct communication between the learner and the performing surgeon, allowing students to have their questions addressed in real-time. Additionally, it enables more students to engage simultaneously, as they can observe the technique on a large screen, eliminating the need for physical proximity often required in open surgeries.

The study aimed to compare undergraduate students' understanding of pelvic retroperitoneal anatomy and their perceptions of an interactive novel teaching method vs traditional classroom instruction.

## Methodology

### Study design

This is a quasi-randomized, prospective Educational Intervention study, employing a parallel group trial design with a superiority framework. The study was conducted between February 15, 2024, and August 30, 2024, at a tertiary teaching institution in western India.

The study commenced following approval from the institutional ethics committee. No: IEC/BU/2024/Ex.04/16/2024. The trial was registered with the Clinical Trials Registry. No: CTRI/2024/02/062476. The study adheres to the most recent principles of the Declaration of Helsinki.^4^ The article conforms to the CONSORT guidelines.^5^ The final academic evaluation of all the students involved in the study was independent of their participation in this study and its results

### Participants

Final year undergraduate students. A consent form was obtained from all students, mentioning their willingness to participate in the program

### Sample size

The entire batch of 152 students was included in the study, resulting in 76 participants per group. This sample size was determined by the structure of the academic schedule rather than through statistical calculation. However, based on power analysis, a minimum of 36 participants per group would have been sufficient to detect a clinically meaningful difference of 2 points, assuming a standard deviation of 3.00, with 80% power and a significance level (α) of 0.05. Thus, our sample size exceeded the required minimum, enhancing the robustness of the study findings.

### Randomization

Owing to academic exigencies, conventional randomization was not feasible. Therefore, the preassigned clinical posting groups of final-year MBBS students—determined in advance by the academic administration—were utilized as units of allocation. Both arms were comparable in their baseline characteristics. The gender distribution in the intervention and control group was comparable (36 boys in intervention & 39 boys in control [*P*>.05]). All the students are of the same year, so age is comparable across groups. Six such predefined groups were randomly assigned to either the intervention or control arm, based on a computer-generated plan, yielding two cohorts of 76 students each. To minimize potential confounding and cross-contamination of outcomes, the uniformity of the predefined clinical batches was maintained.

## Procedure

Members of the research team engaged with the entire study population, encompassing both the intervention and control groups. Each group was guided by dedicated mentors who remained constant throughout the study. The program was conducted as a single-session module, delivered in batches of 10–15 students for both groups.

### Educational tool

Exposure to live demonstration of retroperitoneal dissection with an interactive discussion on vascular and organ anatomy in the OT, emphasizing uterine artery occlusion at the origin. (Video S1) [https://youtu.be/DskDZRo1p1U?si=cfn0DLaxSqnioSgn]

After induction of general anesthesia, a 10 mm port and three 3 mm ports were established.

Dissection began laterally within the triangle of freedom, demarcated by the round ligament anteriorly, the infundibulopelvic ligament medially, and the internal iliac vessels laterally (Figure 1). Entry was achieved by incising either the round ligament or the overlying peritoneum, followed by careful dissection of the areolar tissue to expose the ureter within the medial leaf of the broad ligament. The internal iliac artery was identified, and its uterine branch isolated. The uterine artery was then sealed at its origin from the internal iliac artery using a vessel-sealing device with meticulous traction applied to maintain a safe distance from the ureter. The remainder of the surgery proceeded in accordance with the principles of conventional total laparoscopic hysterectomy

### Control group

The student population in the control group was briefed regarding their role in the program. A pretest questionnaire on the concerned lesson was administered to them. Thereafter, the control group received traditional classroom instruction on the lesson in batches, which included prerecorded online videos.

### Intervention group

The intervention group was informed of their role in the program and then subjected to a pretest. They were then exposed to the novel educational approach in the OT, where the dissection of the retroperitoneal space was demonstrated. Key anatomical structures and their clinical relevance were discussed, and students were encouraged to communicate directly with the operating surgeon. They had no exposure to the classroom instruction.

### Outcomes

A pretest questionnaire on the concerned lesson was administered to the participants of both groups to establish a baseline for assessment.

As part of the evaluation process, a post-test was administered to the students both shortly after their exposure to the traditional and innovative methods and again six months later, to assess both immediate and sustained impacts.

Both groups were subjected to a common pretest and posttest.

The questionnaire set included short-answer questions (SAQs), multiple-choice questions (MCQs), and pictorial recall questions with similar difficulty levels (Figure S2). The total marks scored out of 20 were recorded.

A feedback survey was also conducted to gather the participants' views on the impact of the educational tool, focusing on students' perceptions of both methods. The survey aimed to assess:

○ Whether the method was student-centric, interactive, and informative.
○ Whether the experience enhanced their existing knowledge of the subject matter.
○ How would they recommend applying this method in future medical education?

Additionally, an open-ended question invited participants to share whether they found the experience engaging or refreshing. The questionnaire and feedback form were developed following an extensive literature review and subsequently validated by the department's faculty.

### Statistical plan

Statistical analysis was performed using STRATA 14.4. A parallel group trial design and superiority framework were considered. We aimed to observe an average difference of at least 2 units between the two groups, with the expectation that the intervention group would demonstrate superior performance. A mean change of 2 units was considered both educationally meaningful and practically achievable. To determine the improvement in basic retroperitoneal anatomy after the exercise, the marks of pre- and postexercise were compared. Paired t-test was used to find within-group changes, while an independent sample t-test on difference scores was used to document between-group differences. The perception and acceptability of this novel interactive learning method were evaluated using student feedback. Likert scale responses were analyzed in terms of frequency percentages, while the open-ended responses were examined through qualitative analysis.

## Results

**Figure 2:**
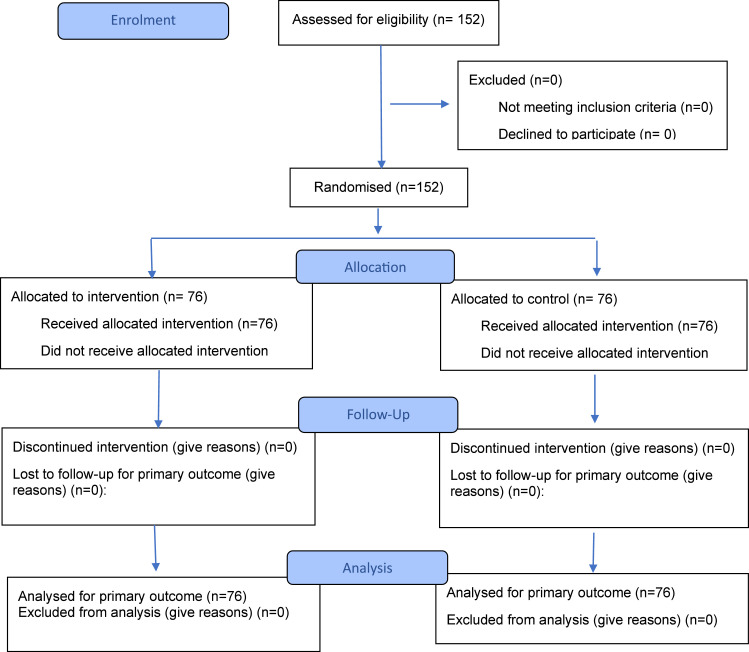
CONSORT flow chart.

The study enrolled 152 final-year MBBS students. Both the intervention and control groups consisted of 76 students each. The flow of participants is shown in the CONSORT flow diagram (Figure 2).

Bhattacharjee et al. In-theatre demonstration of laparoscopic retroperitoneal anatomy as an educational tool for final-year MBBS students. Am J Obstet Gynecol 2025.

**Figure 3:**
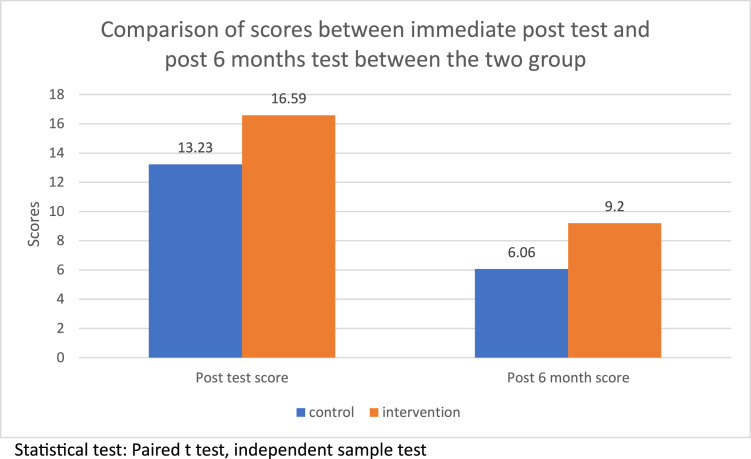
Comparison of scores between the immediate post-test and the post-6-month test between the two groups.

Table presents the baseline performance of both groups before exposure to either method. The conventional group achieved a mean pretest score of 7.50 ± 2.73, while the intervention group scored 7.92 ± 2.37. An independent sample t-test revealed no statistically significant difference between the two groups at baseline. Table further compares the differences between pretest and post-test scores, as well as post-test and six-month follow-up scores, across both groups. The intervention group showed a greater mean improvement from pre- to post-test (8.67 ± 2.81) compared to the conventional group (5.74 ± 3.18). A paired sample t-test with a 95% confidence interval revealed a statistically significant improvement (*P*<.001). The mean difference was 2.93 points (95% CI: 1.96 to 3.90; *P*<.001), and the effect size (Cohen’s *d*) was 0.98, indicating a large effect. The mean difference from post-test to 6 months follow-up was (−7.243±2.168) in the control and (−7.394±1.774) in the intervention group. *P*=.0638 (Table). However, assessments conducted after six months showed a decline in performance to 6.06 ± 1.34 for the conventional group and 9.20 ± 1.36 for the intervention group (Figure 3).

**Table:** Comparison of the mean pretest and the differences of pretest—post-test, and post-test- 6 months post-test between the two groups.

	Group	N	Mean	Std. deviation		*P*-value
Pretest Mean (out of 20)						
	CONTROL	76	7.500	2.728	.3130	.311
	INTERVENTION	76	7.921	2.365	.2713	
DIF_POST_PRE	CONTROL	76	5.743	3.17857	.36461	<.001
	INTERVENTION	76	8.671	2.80660	.32194	
DIF_6M_POST	CONTROL	76	-7.243	2.16871	.24877	.063
	INTERVENTION	76	-7.394	1.77448	.20355	

Statistics: Independent sample test, paired t-test.

Bhattacharjee et al. In-theatre demonstration of laparoscopic retroperitoneal anatomy as an educational tool for final-year MBBS students. Am J Obstet Gynecol 2025.

**Figure 4:**
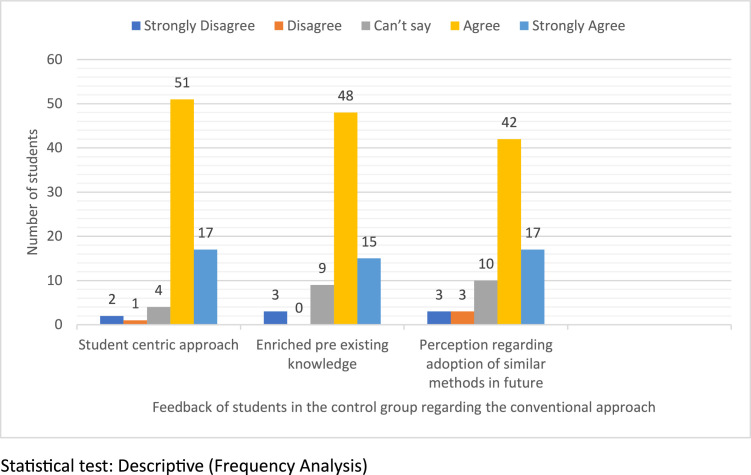
Feedback of students in the control group regarding the traditional approach.

Figure 4 presents student feedback on the conventional teaching method, evaluated using a 5-point Likert scale and reported as percentages. Regarding the method's student-centric nature, 22.7% strongly agreed, 68% agreed, 5.3% were neutral, while 1.3% and 2.7% disagreed and strongly disagreed, respectively. On whether the method enhanced preexisting knowledge, 20% strongly agreed, 64% agreed, 12% were neutral, and 4% strongly disagreed. When asked about adopting similar methods in the future, 32.7% strongly agreed, 56% agreed, 13.3% were neutral, and 4% each disagreed and strongly disagreed.

Bhattacharjee et al. In-theatre demonstration of laparoscopic retroperitoneal anatomy as an educational tool for final-year MBBS students. Am J Obstet Gynecol 2025.

**Figure 5:**
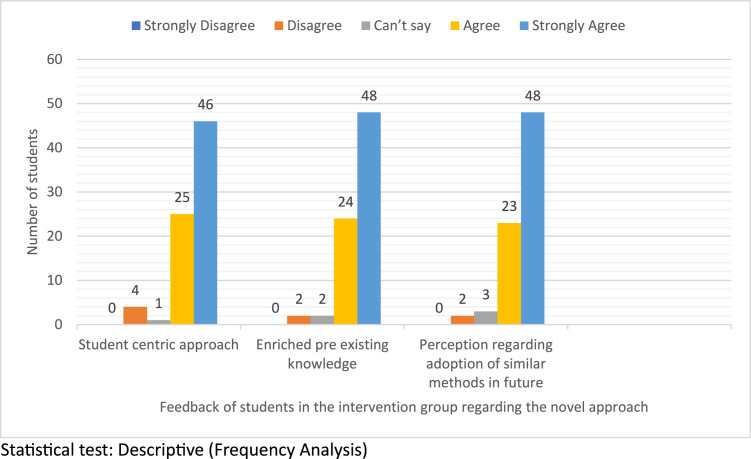
Feedback of students in the Intervention group regarding the novel approach.

Figure 5 illustrates the feedback from students in the intervention group. A majority (60.5%) strongly agreed and 32.9% agreed that the method was student-centric, with only 5.3% disagreeing and 1.3% remaining neutral. On whether the experience enriched their existing knowledge, 63.2% strongly agreed, 31.6% agreed, 2.6% were neutral, and 2.6% disagreed. Regarding the adoption of the tool in future medical education, 63.2% strongly agreed and 30.3% agreed, with minimal neutrality (3.9%) and disagreement (2.6%). Notably, no students selected "strongly disagree," highlighting higher acceptability and enthusiasm for the novel method compared to the traditional approach.

Bhattacharjee et al. In-theatre demonstration of laparoscopic retroperitoneal anatomy as an educational tool for final-year MBBS students. Am J Obstet Gynecol 2025.

Qualitative analysis of the open-ended responses generated a rich collection of observations. The compiled understanding of the feedback from the intervention group revealed overwhelmingly positive feedback. Students described the initiative as a “good initiative” and “one of the best MBBS experiences.” Commonly cited benefits included improved understanding (14 mentions), clarification of concepts (9), and enhanced academic enthusiasm. The interactive, innovative format was praised for delivering in-depth knowledge (5 mentions) and facilitating practical application of theory (5), leading to better anatomical orientation and refined surgical skills (4 each). Sessions were frequently described as “enlightening,” “inspiring,” and “motivating,” with students also calling them “informative” (7 mentions) and “refreshing” (3). The integrated approach was noted to strengthen critical thinking, retention, and confidence (2 mentions each). Overall, students viewed the experience as a student-centric, effective model for future medical education and expressed a strong desire for similar sessions, highlighting the initiative’s significant educational impact.

The feedback from the control group reflects a generally positive reception. Participants frequently described the experience as “informative” (18 mentions) and “enjoyable” (4), citing benefits such as improved understanding (5 mentions), enhanced knowledge (5), and its effectiveness as a visual and long-term memory aid. Many found the method easier to grasp than traditional book-based or didactic approaches. The approach was also seen as “helpful” and “understandable” (2 mentions each), with several highlighting its potential to improve practical knowledge and surgical understanding (2 mentions each). While a few suggested the sessions could be more interactive, others expressed interest in more direct surgical exposure. Overall, the initiative was described as a “great” and “good” experience (2 mentions each), offering better clarification (3 mentions) and reinforcing pre-existing knowledge. A few participants chose not to provide detailed comments.

## Discussion

### Main findings

The present study noted that the pretest scores of both groups were comparable (*P*=.311); however, postintervention, the intervention group attained a mean score of 16.59±1.59 compared to 13.23 ± 2.36 in the conventional group. This demonstrates that students who engaged with the new instructional tool exhibited significantly greater improvement in performance, as evidenced by the mean difference between post-test and pretest scores (8.67±2.806), compared to those who received traditional instruction (5.743±3.178) (*P*=.001). The average difference was 2.93 points, with a 95% confidence interval ranging from 1.96 to 3.90 and a *P*-value of less than .001. The effect size, measured by Cohen’s d, was 0.98, indicating a large effect. At the six-month follow-up, the mean scores declined for both groups. The mean difference between the post-test and 6-month follow-up was (−7.243±2.168) in the control and (−7.394±1.774) in the intervention group. *P*=.0638.

Furthermore, analysis of student feedback revealed a generally positive reception toward both live demonstrations and classroom video sessions across both groups. However, a clear preference emerged for the live, interactive sessions, which students found more engaging and impactful than the traditional system. Notably, to the best of our knowledge, no studies currently exist in the literature evaluating the effectiveness of live demonstrations of retroperitoneal anatomy for undergraduates.

### Interpretation

**Figure 6:**
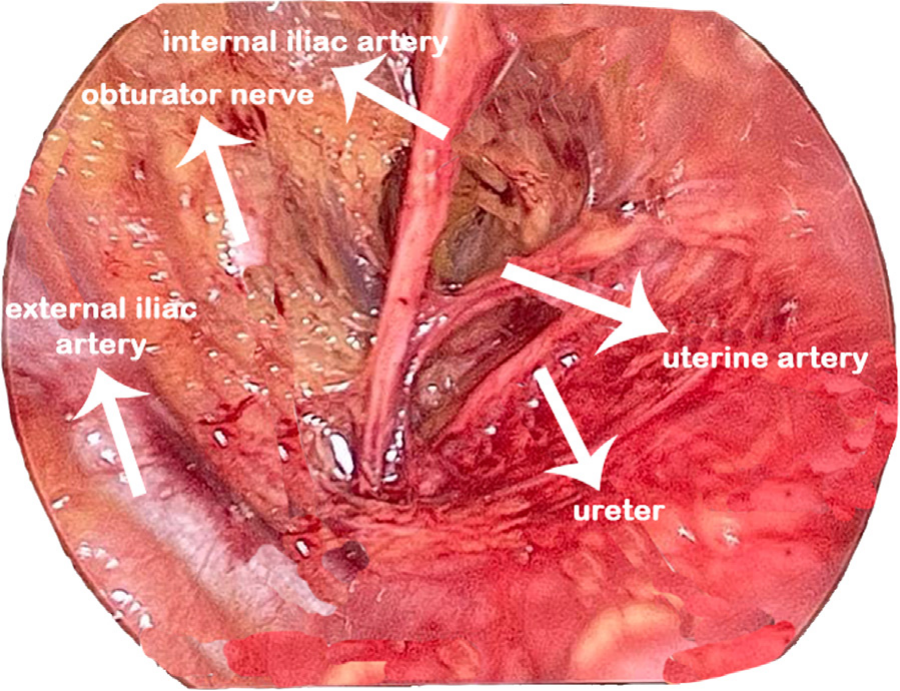
Retroperitoneal dissection.

The retroperitoneal region is a two-dimensional virtual space defined by the round ligament, infundibulopelvic ligament, and external iliac vessels, often referred to as the “triangle of freedom.” This area can be accessed by incising the round ligament or directly cutting the parietal peritoneum overlying the space. Once entered, it allows for direct visualization of the major blood vessels and their branches and the ureter within the medial peritoneal leaf^6^ (Figure 6).

Bhattacharjee et al. In-theatre demonstration of laparoscopic retroperitoneal anatomy as an educational tool for final-year MBBS students. Am J Obstet Gynecol 2025.

The occlusion of the uterine arteries by ligature or coagulation at their origin from the internal iliac results in organ ischemia.^7^^,^^8^ The female pelvis is, in general, a very vascular region. The uterus is primarily supplied by the uterine artery (branch of the anterior division of the internal iliac artery) and the Ovarian artery (branch of the Abdominal Aorta).^6^ Complex pelvic pathology can hinder uterine artery ligation at the conventional site, which is at the level of the isthmus, making alternative approaches, like ligation at the origin, valuable for haemostasis and visualization.^9^ A thorough understanding of pelvic anatomy is essential to navigate the complexities presented by diverse pathological conditions and anatomical variations. The complexity of the pelvic vasculature makes retroperitoneal anatomy challenging for medical students to grasp.^10^ Live interactive laparoscopic demonstrations enhance student engagement, retention, and understanding by linking observation with clinical application.

A study by Van Bonn SM et al found that 61.5% of 65 medical students reported increased knowledge after viewing a live-streamed surgery. Most students (83%) supported integrating live surgeries into medical education, with 64.6% viewing it as a viable alternative to traditional methods. Only 3% had concerns about stream quality, while 89.2% advocated for more frequent live-stream opportunities.^11^ These findings highlight live-streamed surgeries as an effective and engaging supplement to conventional medical teaching.

Kamalakshy J et al compared the effectiveness of live interactive vs prerecorded surgical video demonstrations as teaching methods for cataract surgery among undergraduates. Their study found that the mean marks achieved by students in both groups were comparable (16.26±2.17 in the live group vs 17.03±1.74 in the video group). However, students strongly preferred live teaching in the operating theater.^12^ The operating theatre offers a unique and captivating educational experience for students. Shin et al discovered that a virtual case-based curriculum notably boosted students' self-confidence in independently completing a surgical clerkship program. Scores on a 5-point Likert scale increased from 2.0 before the program to 4.0 afterward (*P*=.0001).^13^

The present study revealed an important finding: the mean scores of students in both groups were lower after six months compared to their performance immediately following the exposure. Although both groups experienced a decline in performance over time, the intervention group still performed better than the control group. Similarly, Pooja H et al found that students using audiovisual aids scored higher on immediate MCQ tests than those using text alone. Although scores declined in both groups after four weeks, the audiovisual group maintained better performance.^14^

The human cognitive architecture comprises short-term working memory and long-term memory. To facilitate the retention of knowledge in long-term memory, it is recommended that the capacity of working memory be enhanced through frequent use of audiovisual aids and interactive sessions. Repetition is crucial for both retention and concept formation.^15^^,^^16^

French et al, in their study, cite Anna Sfard’s “two metaphors” model, which posits that learning is maximized when it integrates both acquisition and participation. While spaced repetition is invaluable for consolidating large volumes of information, it is less suited to the assimilation of novel concepts.^17^ In this context, blended learning warrants further attention. Vallée et al, in their systematic review and meta-analysis, demonstrated that comprehension in medical education is consistently superior with blended learning compared to traditional methods.^18^ In their study, Prem Kumar et al found that among various teaching methods, audiovisual aids were linked to better memory retention. However, participatory methods such as role-play, case studies, and microteaching yielded the best outcomes for students and health professionals. He also noted that brainstorming activities must be conducted more frequently to achieve optimal results for consolidating information and sustaining long-term memory.^19^

Glossop et al conducted a systematic review of undergraduate surgical skills training in the UK and found that medical students consistently fall short of meeting the General Medical Council’s minimum requirements for basic surgical competencies. This shortfall within the undergraduate curriculum may serve as a significant deterrent, potentially discouraging students from pursuing a career in surgery.^20^

### Clinical implications

Technology has transformed surgical education, highlighting the need for platforms that enhance the learning of complex skills. The study showed that live interactive sessions promoted active learning, boosting attention, motivation, and satisfaction, thus building a strong foundation for future surgical skill development. The application of these techniques, as an educational tool, warrants further research investment.

### Research implications

This innovative approach has the potential to profoundly influence the current educational system, fostering positive metacognitive behaviour and enhancing problem-solving skills among learners. Integrating such models into the teaching curriculum could sustain learners' engagement, thus producing competent and confident doctors equipped to serve the community effectively.

## Strengths and limitations

The main strength of this study is that it is among the first to assess live, interactive laparoscopic demonstrations focused on retroperitoneal anatomy for undergraduates, addressing a lacuna in the literature. A large sample size and validated assessment tools enhanced validity. While not fully randomized, using preassigned groups minimized cross-contamination and supported real-world feasibility. The study combined pre-/post-tests with qualitative and quantitative feedback, ensuring a comprehensive evaluation. Appropriate statistical methods ensured rigor, and a six-month follow-up added insight into knowledge retention.

A key limitation of this study is the use of preassigned clinical groups, which may have introduced allocation bias and reduced internal validity as compared to a randomized design. Conducted at a single tertiary institute, generalizability to nontertiary or nonurban settings is limited. Awareness of group allocation by students and instructors may have introduced performance and observer biases. The study did not account for prior surgical exposure, baseline interest, or other academic factors that may have influenced outcomes. While theoretical knowledge improved, practical surgical or anatomical skills were not assessed. Qualitative feedback was subjective and potentially biased by social desirability or the novelty of the intervention.

## Conclusions

This study examined the role of teachers as facilitators in conveying surgical and anatomical knowledge to students. It found that live interactive sessions in the operating theatre were more student-centric, effectively captured attention, promoted active learning, facilitated doubt clarification, and enhanced topic retention. We believe our study offers a novel and impactful educational strategy that enhances anatomical learning and clinical competence.

## Data availability

The datasets generated during and/or analyzed during the current study are available from the corresponding author upon reasonable request.

## Study design

Quasi-Randomized trial, Educational intervention tool

## Ethical considerations

This is an educational interventional study, and no harm is anticipated to any participants. The study commenced following approval from the institutional ethics committee. No: IEC/BU/2024/Ex.04/16/2024. A consent form was obtained from all students, mentioning their willingness to participate in the program.

• The authors declare that there is no conflict of interest and no funding was obtained.
• The manuscript has been read and approved by all the authors, and all authors meet the authorship criteria. This is an original and honest work.
• The study conforms to the latest Helsinki Declaration norms.
• All authors accept responsibility for the paper as published.

## Reporting guidelines

The article adheres to the CONSORT guidelines.

## Data availability statement

The datasets generated during and/or analyzed during the current study are available from the corresponding author upon reasonable request.

## CRediT authorship contribution statement

**Rumi Bhattacharjee:** Writing – review & editing, Writing – original draft, Visualization, Validation, Supervision, Software, Resources, Project administration, Methodology, Investigation, Funding acquisition, Formal analysis, Data curation, Conceptualization. **Sangita Pandey:** Writing – review & editing, Validation, Supervision, Resources, Project administration, Methodology, Investigation, Funding acquisition, Formal analysis, Data curation, Conceptualization. **Manisha Jana:** Writing – review & editing, Writing – original draft, Visualization, Validation, Resources, Methodology, Investigation, Funding acquisition, Data curation. **Jaishree Ganjiwale:** Writing – review & editing, Visualization, Validation, Software, Resources, Project administration, Methodology, Investigation, Formal analysis, Data curation. **Nitin Raithatha:** Writing – review & editing, Visualization, Validation, Supervision, Resources, Project administration, Methodology, Investigation, Funding acquisition, Formal analysis, Data curation. **Somashekhar Nimbalkar:** Writing – review & editing, Visualization, Validation, Supervision, Resources, Project administration, Methodology, Investigation, Formal analysis, Data curation.

## Appendix Supplementary materials

Supporting information It can be found online in the relevant section. Video S1: Retroperitoneal Dissection with uterine artery occlusion at origin. Figure S2: Questionnaire (MCQs & SAQs). Image, application 1

Image, video 2
