# Supplementary material for: In-theatre demonstration of laparoscopic retroperitoneal anatomy as an educational tool for final-year MBBS students: a novel quasi randomized experiment
Source: AJOG Glob Rep. 2025 Oct 9;5(4):100572. doi: 10.1016/j.xagr.2025.100572 (PMC12634837; doi:10.1016/j.xagr.2025.100572)
Supplement: Supplementary file 1 — Supporting information It can be found online in the relevant section. Video S1: Retroperitoneal Dissection with uterine artery occlusion at origin. Figure S2: Questionnaire (MCQs & SAQs). [file mmc1.docx]

Figure S2: Questionaire ( MCQs & SAQs)


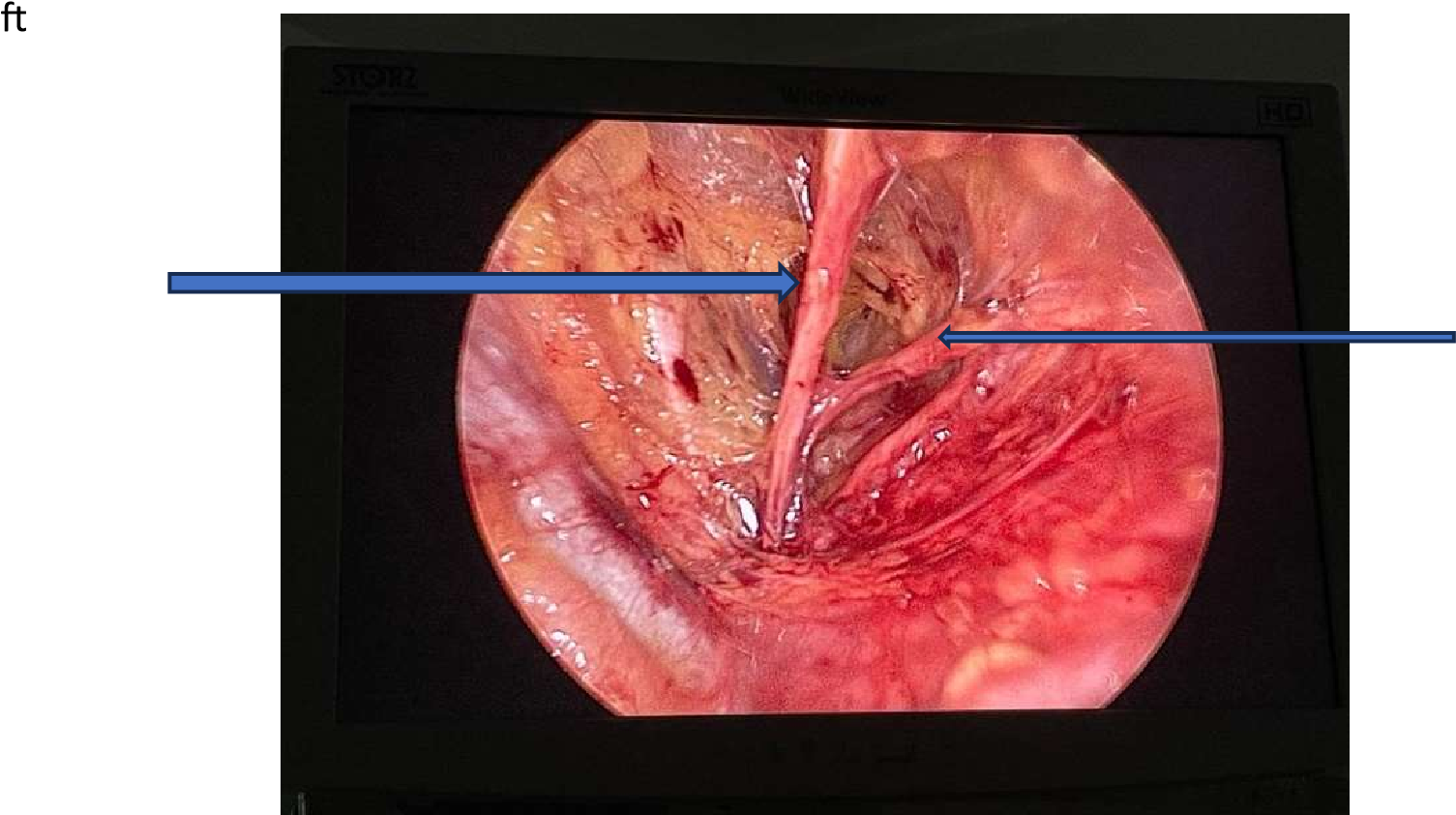
1. Image of the left pelvic wall

Q1. In this image, identify the 2 structures marked by arrows ( 1 mark )

Image of the left lateral pelvic wall


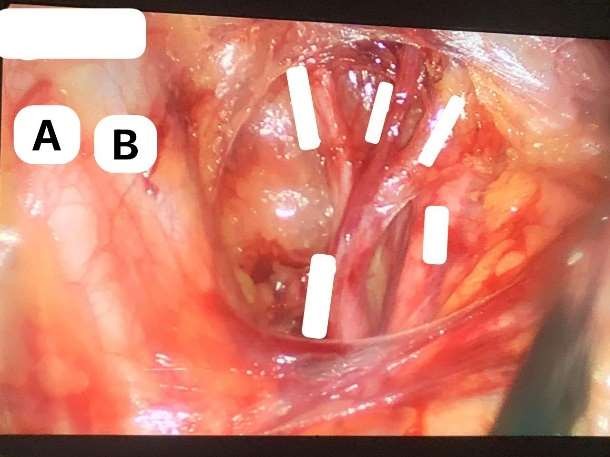


Q2. In this image, identify the structures marked A and B. (1 mark)

3. The anatomic landmark for the division of the aorta into the right and left common iliac arteries is

(i) S1, S2 (ii)L5,S1 (iii)L3, L4 (iv) L 4,L5 ( 1 mark)

4. Mention the two main vessels that supply blood to the uterus (1 mark)

5. The first step in accessing the retroperitoneum is (1 mark )

(i) An Incision over the lateral parietal peritoneum between the round ligament and the infundibulopelvic ligament

(ii)An Incision anterior to the round ligament

(iii)An Incision over the infundibulopelvic ligament

(iv)An incision between the fallopian tubes and the round ligament

6. Which nerve runs upon the belly of the psoas major muscle, and why should an injury to this nerve be avoided during dissection of the retroperitoneum? (2 marks)

7.The ureter is identified ( 1 mark)

1. On the base of the broad ligament, in its medial leaf
2. At the apex of the broad ligament
3. At the apex of the pubovesicocervical ligament
4. On the base of the round ligament

8. Which one of the following statements is correct (1 mark)

(i)The ureter enters the pelvis over the division of the internal iliac arteries

(ii)The pelvic ureter lies postero-medial to the uterosacral ligaments

(iii)It traverses the cardinal ligament approximately 4-5 cm lateral to the cervix

(iv)The pelvic ureter lies medial to the internal iliac vessels.

9. An injury to which of the following structures is more dangerous and difficult to manage during retroperitoneal dissection (1 mark)

i. Damage to the external iliac artery ii. Damage to the external iliac vein

10. Where should the internal iliac artery be ligated from its bifurcation from the common iliac artery? (1 mark)

(i)At a distance of 0 cm

1. At a distance of 2cm
2. At a distance of 5 cm
3. At a distance of 7 cm
4. 1^st^ medial branch of the internal iliac artery is (1 mark)

(i)Uterine (ii) Middle rectal (iii) internal pudendal (iv) superior vesical

1. State the significance of the Tunnel of Wertheim (2marks)

13. What suture material/ technique is NOT used in ligation of the uterine artery at its origin(1 mark)

1. 1-0 delayed absorbable suture [Vicryl]
2. coagulated using bipolar diathermy
3. stapled with a vascular endoscopic stapler
4. 1-0 chromic catgut

14. The medial umbilical ligament is the terminal end of (1mark)

i.Obliterated umbilical artery ii.Superior vesical iii.Internal pudendal iv.Uterine artery

15. What is the surgical benefit of ligation of the uterine artery at its origin (4marks)

Feedback

Acceptability and perception of students towards a new method of teaching in obstetrics and gynaecology

1. Was this learning experience a welcome/ refreshing one for you?

State the reasons

1. Please encircle the answer of your choice

1. The method was student-centric, interactive, and informative

(i) strongly disagree (ii) disagree (iii) can’t say (iv) agree (v) strongly agree 2. Did this direct visual impact enrich your pre-existing knowledge?

(i) strongly disagree (ii) disagree (iii) can’t say (iv) agree (v) strongly agree

3. How do you recommend the use of such teaching methods in the field of medical education in the future

(i) strongly disagree (ii) disagree (iii) can’t say (iv) agree (v) strongly agree
